# Identification of Fat Mass and Obesity Associated (FTO) Protein Expression in Cardiomyocytes: Regulation by Leptin and Its Contribution to Leptin-Induced Hypertrophy

**DOI:** 10.1371/journal.pone.0074235

**Published:** 2013-09-03

**Authors:** Xiaohong Tracey Gan, Ganjian Zhao, Cathy X. Huang, Adrianna C. Rowe, Daniel M. Purdham, Morris Karmazyn

**Affiliations:** Department of Physiology and Pharmacology, The University of Western Ontario, London, Ontario, Canada; Temple University, United States of America

## Abstract

The recently-identified fat mass and obesity-associated (FTO) protein is associated with various physiological functions including energy and body weight regulation. Ubiquitously expressed, FTO was identified in heart homogenates although its function is unknown. We studied whether FTO is specifically expressed within the cardiac myocyte and its potential role pertaining to the hypertrophic effect of the adipokine leptin. Most experiments were performed using cultured neonatal rat cardiomyocytes which showed nuclei-specific FTO expression. Leptin significantly increased FTO expression which was associated with myocyte hypertrophy although both events were abrogated by FTO knockdown with siRNA. Administration of a leptin receptor antibody to either normal or obese rats significant reduced myocardial FTO protein expression. Responses in cardiomyocytes were accompanied by JAK2/STAT3 activation whereas JAK2/STAT3 inhibition abolished these effects. Expression of the cut-like homeobox 1(CUX1) transcriptional factor was significantly increased by leptin although this was restricted to the cathepsin L-dependent, proteolytically-derived shorter p110CUX1 isoform whereas the longer p200CUX1 protein was not significantly affected. Cathepsin L expression and activity were both significantly increased by leptin whereas a cathepsin L peptide inhibitor or siRNA specific for CUX1 completely prevented the leptin-induced increase in FTO expression. The cathepsin L peptide inhibitor or siRNA-induced knockdown of either CUX1 or FTO abrogated the hypertrophic response to leptin. Two other pro-hypertrophic factors, endothelin-1 or angiotensin II had no effect on FTO expression and FTO knockdown did not alter the hypertrophic response to either agent. This study demonstrates leptin-induced FTO upregulation in cardiomyocytes *via* JAK2/STAT3- dependent CUX1 upregulation and suggests an FTO regulatory function of leptin. It also demonstrates for the first time a functional role of FTO in the cardiomyocyte.

## Introduction

The function of the recently identified fat mass and obesity-associated (FTO) protein is not well-understood although it is likely involved in various aspects of physiological and pathophysiological functions. Some of these include regulation of energy intake and metabolism, adipogenesis, DNA methylation and FTO has also been suggested to act as a transcriptional factor [1], [2]. Global deletion of this nuclear protein in mice leads to profound weight loss [3] whereas a number of genetic variants in FTO predisposes individuals to the development of obesity [4]–[6]. Overexpression of FTO in mice leads to obesity and the accompanying increase in adiposity [7]. Thus, there is a close association between FTO and body mass index. Although FTO is extensively expressed in the brain (where it regulates energy expenditure) it is also expressed in many other tissues including the heart, albeit at substantially lower levels [8]. This suggests that FTO may also regulate organ-specific functions although the nature of the potential roles of FTO in regulation of organ function are completely unknown. Indeed, originally FTO was considered as “a gene of unknown function in an unknown pathway” [4].

The effect of FTO on body weight, adipogenesis and energy metabolism is suggestive of a possible cross-talk between FTO and leptin, a pro-satiety adipokine produced primarily by white adipocytes but also by the cardiomyocyte and whose plasma concentrations are closely correlated to the degree of adiposity [9]. It is interesting that FTO has been shown to colocalize with the long form of the leptin receptor (OBRb, also referred to as LepRb) in the hypothalamus and leptin has been shown to decrease FTO protein levels [10]. To our knowledge, the only other potential association between FTO and leptin thus far reported is a reduction in circulating plasma levels of leptin in mice overexpressing FTO, although the authors considered this as secondary to the increased adiposity [7]. Leptin produces diverse effects on cardiac function and morphology and has been shown to directly produce cardiomyocyte hypertrophy under both *in vitro* and *in vivo* conditions [11]–[13]. Based on the identification of FTO in the heart as well as the initial evidence of interaction between FTO and leptin, the primary goals of the present study were to determine the identification of FTO in cardiomyocytes, assess the effect of leptin on FTO expression and determine the potential role of FTO in leptin-induced cardiomyocyte hypertrophy.

## Methods

### Ethics Statement

All protocols for the use of animals are in accordance with the University of Western Ontario animal care guidelines. These protocols conform to the guidelines of the Canadian Council on.

Animal Care (Ottawa, ON, Canada) and the Guide for the Care and Use of Laboratory Animals published by the US National Institutes of Health (NIH Publication No. 85-23, revised 1996). The study has been approved by the University of Western Ontario Council on Animal Care (protocol number 2009–020).

### Animals

Rats for neonatal ventricular myocyte studies were bred in the Health Sciences Animal Care Facilities at the University of Western Ontario. Adult male Sprague-Dawley rats were purchased from Charles River Canada (St. Constant, Quebec, Canada), and maintained in the Animal Care Facilities in accordance with the guidelines of the Canadian Council on Animal Care (Ottawa, Ontario, Canada).

### Experimental Protocol for Myocyte Culture Studies

Neonatal cardiomyocytes were prepared from hearts of 1- to 3-day-old Sprague-Dawley rats. Ventricles were washed, minced with scissors, and subsequently digested with collagenase II. The reaction was terminated with the addition of 20% FBS. The cellular extracts were strained and centrifuged at 514 *g* for 5 min. The supernatant was then aspirated and the resulting pellet was resuspended in cell culture media. The suspended cell mixture was subjected to two cycles of preplating to eliminate fibroblasts and other nonmyocyte components. The final myocyte preparation was plated in cell culture media containing Dulbecco’s modified Eagle medium/Ham’s F-12 supplemented with 10% FBS, 10 µg/ml transferrin, 10 µg/ml insulin, 10 ng/ml selenium, 50 U/ml penicillin, 2 mg/ml BSA, 5 µg/ml linoleic acid, 3 mM pyruvic acid, 0.1 mM minimum essential medium nonessential vitamins, 10% minimum essential medium vitamin solution, 0.1 mM bromodeoxyuridine, 100 µM L-ascorbic acid, and 30 mM HEPES, pH 7.2. The number of myocytes of each set was counted using a Countess® Automated Cell Counter (Life Technologies Inc, Burlington, Ontario, Canada) in order to achieve a final cell concentrations of 10^6^/ml.

For studies involving pharmacological modulations the following drugs were used: the rat leptin receptor antagonist SHLA (100 ng/ml, Protein Laboratories Rehovot, Israel), the Janus kinase 2 (JAK2) inhibitor AG490 (50 µM, Santa Cruz Biotechnology Inc., Santa Cruz, CA), the Signal Transducer and Activator of Transcription 3 (STAT3) inhibitor S31–201 (10 µM, Santa Cruz) and a cathepsin L inhibitor peptide (10 µM. Sigma-Aldrich Canada Co, Oakville, Ontario, Canada). For each study, the respective agent was added 30 min before leptin (3.1 mM) administration. Additional studies were done to compare the effect of leptin with two other pro-hypertrophic agents, endothelin-1 (10 nM, Sigma-Aldrich) or angiotensin II (100 nM, Sigma-Aldrich). For all experiments, myocytes were exposed to leptin, endothelin-1 or angiotensin II for 24 hours.

### Cell Transfections

We employed a small interfering RNA (siRNA) approach to knockdown either FTO or cut-like homeobox 1 (CUX1) gene expression in cardiac cells. Following appropriate plating, myocyte transfections were performed as recommended by the manufacturer. siRNA was designed to target the FTO gene with the oligonucleotide sequence 5′-CAACGTGACTTTGCTAAACTT-3′ corresponding to nucleotides 588–608 of rat FTO (GenBank accession no. NM_001039713). FTO knockdown was performed by transfecting cells with a pair of siRNA constructs specific for FTO mRNA (5′-ACGUGACUUUGCUAAACUUTT-3′ and 5′-AAGUUUAGCAAAGUCACGUTG-3′). siRNA targeted the CUX1 gene with the oligonucleotide sequence 5′-AGGCCGATGAAACAAATGCAA-3′ corresponding to nucleotides at 2270–2290 of rat CUX1 (GenBank accession no: XM-001070410). CUX1 knockdown was performed by transfecting cells with a pair of siRNA constructs specific for CUX1 mRNA (5′-GCCGAUGAAACAAAUGCAATT-3′ AND 5′-UUGCAUUUGUUUCAUCGGCCT-3′). All siRNA was purchased from QIAGEN Inc. (Mississauga, Ontario. Canada). Each siRNA was transfected into cells with HiPerFect reagent (QIAGEN) for 48 hours. A universal scrambled negative control siRNA (QIAGEN) was used for control studies. The silencing efficacy was confirmed by both AF488-tagged control siRNA and Western blotting at various time points after transfection. Transfection efficiency determined by immunofluorescence analysis ranged between 75–85%.

### Immunoflurescence Determination

Myocytes were grown on collagen-pre-coated glass coverslips in medium. Following 24 hour incubation with treatments, cells were fixed in 4% paraformaldehyde for 20 min at 40C. After 5 min permeabilization in 0.1% Triton X-100 PBS, myocytes were blocked for 1 h in 1% BSA in PBS buffer and then incubated overnight with the primary antibody against either FTO (1∶50 dilution), CUX1 (1∶500) or cathepsin L (1∶100) in 1% BSA in PBS buffer, then for 1 hour with a secondary antibody (AF488-conjugated anti-mouse, Life Technologies). Nuclei were counterstained with the nuclear stain Hoechst 33342 (1 µg/ml, Sigma-Aldrich) for 5 min. Cardiomyocytes were visualized using a Zeiss Axio Observer D1 fluorescence microscope (Zeiss, Gottingen, Germany) and quantification performed using SigmaScan Pro v.5 software (Systat Software Inc., San Jose, CA, USA).

### Myocyte Surface Area Determination

Cell surface area was assessed using a Leica inverted microscope equipped with an Infinity 1 digital camera at ×200 magnification and measured using SigmaScan Software (Systat Software Inc., Chicago, Il). At least 50 randomly selected cells per experiment were used to determine cell size and averaged to provide an N value of one.

### RNA Isolation, Reverse Transcription and Real-Time PCR Analysis

RNA was extracted using Trizol (Life Technologies) according to the manufacturer’s instructions. RNA (1 µg) was used to synthesize the first strand of cDNA using M-MLV reverse transcriptase according to the manufacturer’s protocols and was used as a template in the PCR reactions. The expression of JAK2, STAT3, cathepsin L, FTO, CUX1, ANP, 18S rRNA (loading internal control) genes was determined in 10 µl reaction volumes using EvaGreen qPCR Mastermix (Applied Biological Materials, Richmond, British Columbia, Canada). Fluorescence was measured and quantified using a DNA Engine Opticon 2 System (Bio-Rad Laboratories, Mississauga, Ontario, Canada). Primer sequences for all genes are shown in Table 1.

**Table 1:** Primer oligonucleotide sequences for genes of interest.

Gene	Forward primer sequence	Reverse primer sequence
ANP	5′-ctgctagaccacctggagga-3′	5′-aagctgt-tgcagcctagtcc-3′
β-MHC	5′-gcactggccaagtcagtgta-3′	5′-cgaacatgtggtggttgaag-3′
Cathepsin L	5′- gagagcagtgtgggagaaga-3′	5′-atctgcagcatcagaggttc-3′
CUX1	5′-ggaggaagctgagcacaaac-3′	5′-gaggccagttggagagagtg-3′
FTO	5′- agggctgcaccatcaattac-3′	5′- tcacgttgtaggctgctctg-3′
JAK2	5′-agatatgcaagggcatggag-3′	5′-gtcaaggattcgggagcata-3′
STAT3	5′-tcacttgggtggaaaaggac-3′	5′-tgggaatgtcagggtagagg-3′
18s	5′-gtatcccgttgaaccccatt-3′	5′-ccatccaatcggtagtagcg-3′

### Western Blotting

Myocytes were washed with PBS and lysed with 100 µl of lysis buffer following homogenization, and centrifuged at 10 000 g for 5 minutes at 4°C. The supernatant was transferred to a fresh tube and the protein concentration was determined by the Bradford protein assay method (BioRad). Thirty micrograms of protein were resolved on a 10% SDS-polyacrylamide gel and transferred to nitrocellulose membranes. The membranes were blocked in 5% milk for 1 hour and incubated with primary antibodies for FTO (Cayman Chemicals, Ann Arbor, MI, 1∶500 dilution), p200CUX1 (Santa Cruz or Abcam, Toronto, Ontario, Canada, 1∶500), p110CUX1 (Thermo Scientific, Rockford, Il, 1∶500) cathepsin L (LifeSpan BioSciences, Seattle, WA,1∶500), JAK2 (Santa Cruz, 1∶1000), STAT3 (Santa Cruz, 1∶1000) or β-actin (EMD Millipore, Billerica, MA, 1∶1000) overnight, followed by either a secondary antibody for 1 hour or by adding the IRDye 800-conjugated secondary antibody (1∶5000; LI-COR Biosciences, Lincoln, NE) to the primary antibody. The bound complex was then detected either by enhanced chemiluminescence reagent (Amersham Biosciences Inc., Piscataway, NJ) or using the Odyssey Clx Infrared Imaging System (Li-COR). The images were analyzed for density using the Odyssey Application Software (Li-COR).

### Cathepsin L Activity Assay

Cathepsin L activity in cell lysates was assayed using a Cathepsin L Activity Fluorometric Assay Kit according to the manufacturer’s instructions (BioVision Inc, Milpitas, CA). This is a fluorescence-based assay that utilizes the preferred cathepsin-L substrate sequence FR labeled with AFC (amino-4-trifluoromethyl coumarin). Cell lysates that contain cathepsin-L cleave the synthetic substrate FR-AFC to release free AFC. Fluorescence intensity was determined at excitation/emission wavelengths of 400 nm/505 nm using a Spectramax M5 spectral fluorimeter (Molecular Devices, Sunnyvale, CA).

### In Vivo Studies

Experiments were performed to determine if endogenous leptin regulates cardiac FTO levels in normal rats or rats made obese by a high fat diet. For these studies adult male Sprague-Dawley rats weighing 225 to 250 g were fed either a standard diet or a high fat diet for 12 weeks. The breakdown of calories in the standard diet was 17% from protein, 73% from carbohydrate and 10% from fat. In the high fat diet 17% of calories were from protein, 35% from carbohydrate and 48% from fat (Research Diets, New Brunswick, NJ). To examine the role of leptin in regulating cardiac FTO protein expression animals from each dietary group were injected with leptin receptor antibody (OBR-Ab) (4 ug/kg bw) every other day for the duration of the 12 week feeding period. This dose of OBR-Ab was previously found to prevent leptin-induced cardiac STAT activation [14]. At the end of the 12 week period the animals were sacrificed by decapitation and hearts removed for FTO analysis by Western blotting.

### Statistical Analyses

Results are presented as means ± standard error. The data were analyzed by a 1-way ANOVA and group differences were detected using a Student-Newman-Keuls post hoc test when initial ANOVA analysis revealed statistically significant differences. P values of <0.05 were considered significant.

## Results

### Expression of FTO in Cardiomyocytes and its Up-Regulation by Leptin

**Figure 1:**
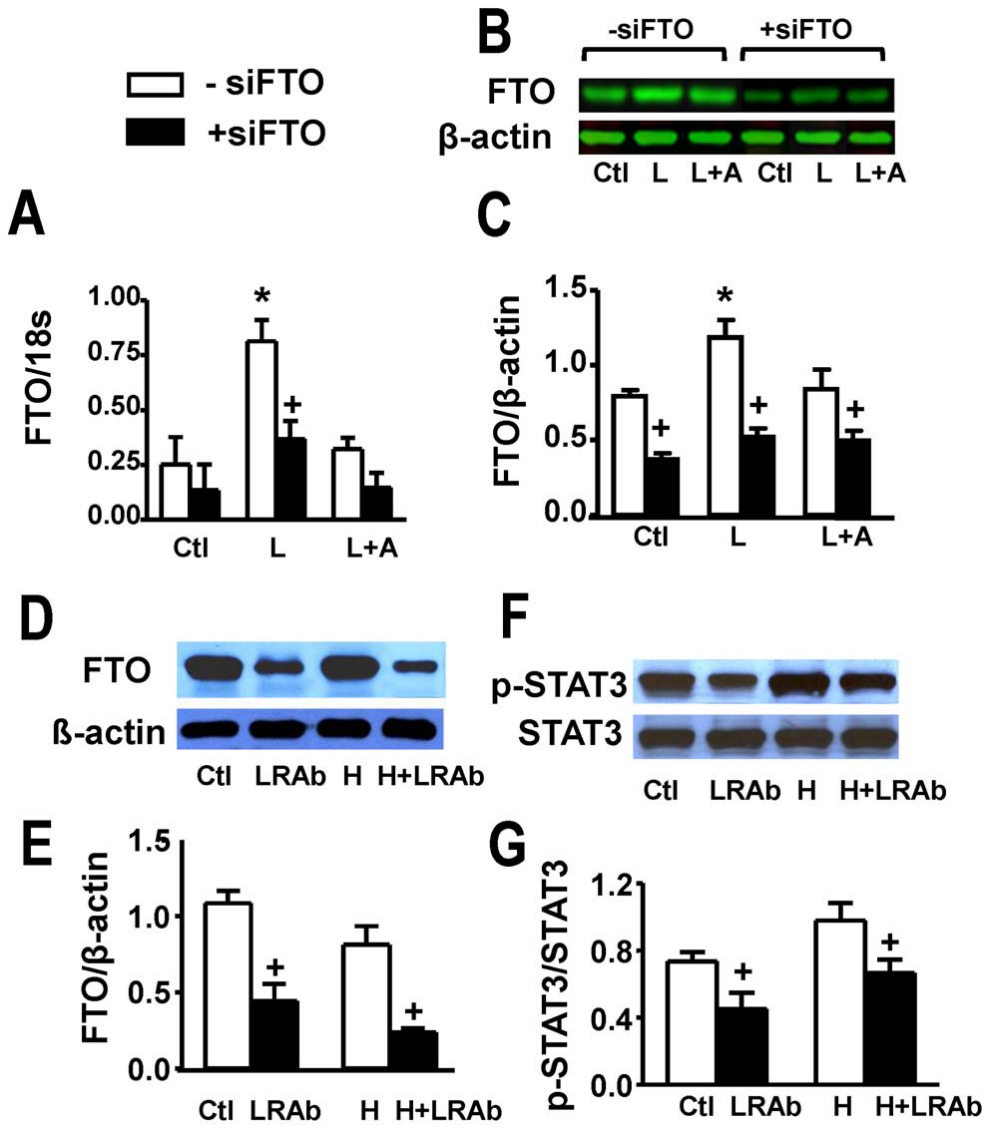
Identification of FTO in cardiomyocytes and cardiac tissue and evidence for its upregulation by leptin. Panel A shows FTO gene expression in cultured rat ventricular myocytes under control conditions (Ctl) and its stimulation by leptin (L) and lack of effect in the presence of the leptin receptor receptor antagonist SHLA (A). Panels B and C show Western blots and their quantitative analyses with identical treatments as shown in panel A. These panels also show suppression of FTO in myocytes transfected with FTO siRNA. Panels D and E demonstrate Western blots and their quantitative assessment of whole hearts from control animals or animals fed a high fat diet (H) for 12 weeks. The results also show suppression of cardiac FTO protein expression in animals treated with an antibody directed against the leptin receptor (LRAb) every two days during the feeding period. Panels F and G show identical treatments as for panels D and E demonstrating inhibition of STAT3 phosphorylation in hearts of animals treated with the LRAb. Quantitative data are presented as mean+SEM. N = 8 for myocyte data in panels A and C and N = 5 for data shown in panels E and G. *P<0.05 from respective control group (or H group in panels E and G); ^+^P<0.05 from respective group in the absence of siFTO (-siFTO).

Our first set of experiments was aimed to identify the presence of FTO in cultured myocytes and determine whether this can be modulated by leptin treatment. Figure 1 summarizes our results in which FTO gene expression was determined as well as protein levels assessed by Western blotting. As evident from Figure 1A, 24 h leptin treatment resulted in a three-fold increase in FTO gene expression. Moreover, Western blot analysis (Figure 1B and 1C) showed substantial abundance of FTO in myocytes as well as the ability of 24 h leptin treatment to produce approximately a two-fold elevation in FTO levels. These data also demonstrate that siRNA directed at FTO substantially decreased FTO protein levels (Figure 1B and 1C). Although leptin tended to increase FTO gene and protein expression in siFTO-treated myocytes no significant effect of leptin was seen under this condition. Moreover, all effects of leptin on FTO were, as would be expected, completely abrogated by the highly potent and specific leptin receptor antagonist SHLA.

The ability of leptin to potently increase FTO levels in cultured myocytes prompted us to examine whether endogenous leptin regulates cardiac FTO levels *in vivo* in normal rats as well as in rats made obese by high fat diet consumption for 12 weeks. Body weights for control animals at the end of the feeding period were 623±9 g whereas for rats fed a high fat diet mean body weights were 781±19 g (P<0.05), representing approximately a 25% increase. Moreover, plasma leptin levels in these animals were nearly doubled from 5.3±1.1 ng/mL in control animals to 10.3±0.8 ng/mL (P<0.05) in animals treated with the high fat diet. As shown in Figure 1D and 1E, the high fat diet and the accompanying increase in both body weight and plasma leptin concentrations had no effect on cardiac FTO protein expression. However, treatment with an antibody directed against the leptin receptor, at a dose which significantly inhibited STAT3 activation (Figure 1F and 1G), resulted in a marked and significant decrease in cardiac FTO levels in both dietary groups (Figure 1D and 1E) although body weights were not significantly affected (not shown).

**Figure 2:**
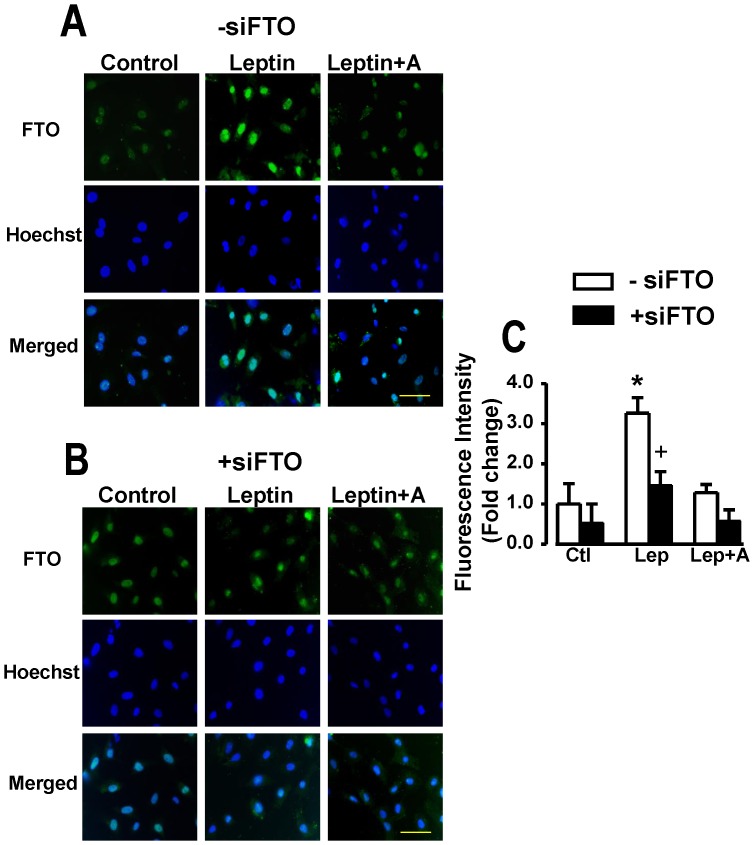
Immunofluorescence identification of FTO in cardiomyocytes. Panel A demonstrates immunofluorescence images and their quantitative assessment demonstrating nuclear localization of FTO in cardiomyocytes and its upregulation by leptin whereas panel B shows identical data in myocytes transfected with siFTO illustrating suppression of FTO. Corresponding images of myocytes stained with the nuclear dye Hoechst 33342 as well as merged images are also presented. Quantitative data in panel C are presented as mean+SEM, N = 8. *P<0.05 from respective control (Ctl) group; +P<0.05 from respective group in the absence of siFTO (−siFTO). Lep, leptin; A, leptin receptor antagonist. Horizontal bar in bottom right images indicates 200 µm.

As shown in the immunofluorescence images (Figure 2), FTO expression under control conditions as well as in myocytes treated with leptin was restricted to nuclei with FTO intensity increasing more than three-fold by leptin in those myocytes not treated with siFTO (Figure 2A and 2C)). However, myocytes treated with siFTO demonstrated significantly reduced FTO fluorescence intensity with no significant increase in response to leptin (Figure 2B and 2C). The ability of leptin to upregulate FTO expression within nuclei was abrogated by the leptin receptor antagonist (Figure 2A and 2C).

### Lack of Role of FTO in Mediating Endothelin-1 or Angiotensin II Induced Cardiomyocyte Hypertrophy

**Figure 3:**
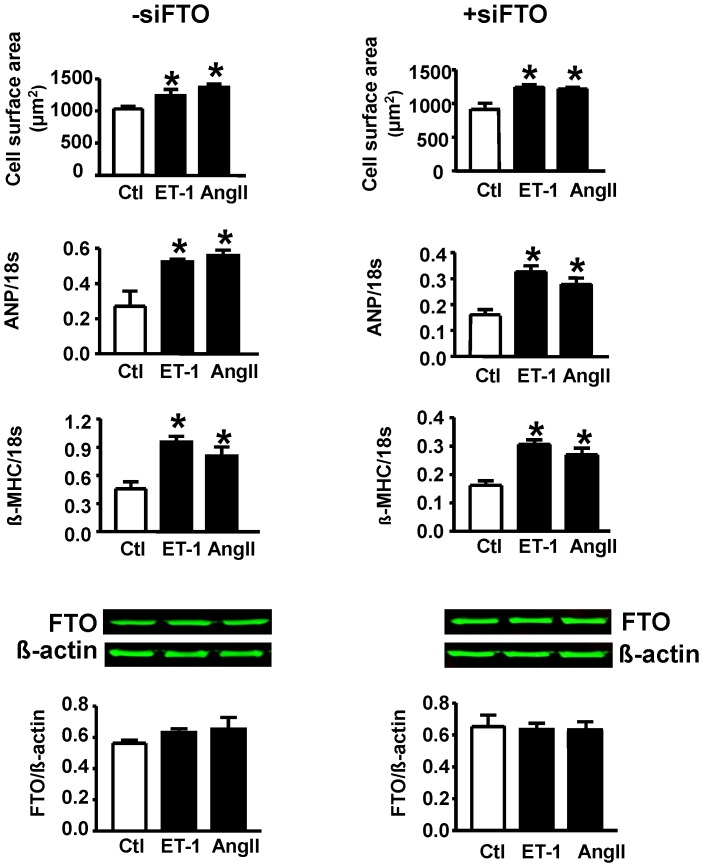
Hypertrophic responses to either endothelin-1 (ET-1) or angiotensin II (Ang II) in the absence (−siFTO) or presence (+siFTO) of FTO downregulation. Hypertrophy was assessed by determining cell surface area as well as expression of ANP or β-MHC. Bottom two panels show Western blot data with representative blots for different treatments. N = 5. Ctl, control. No differences were observed between any treatment group.

Figure 3 (top six panels) summarizes the responses of myocytes to either endothelin-1 or angiotensin II in terms of FTO expression as well as hypertrophy and the latter following FTO downregulation. Both endothelin-1 and angiotensin II produced a significant hypertrophic response as evidenced by increased myocyte surface area as well as both atrial natriuretic peptide (ANP) and β- myosin heavy chain (β-MHC) gene expression although these responses were unaffected by siRNA directed at FTO. As also evident from Figure 3 (bottom two panels) neither endothelin-1or angiotensin II had any effect on FTO protein expression.

### FTO Upregulation: Dependence on JAK2/STAT3 and CUX1 Activation

**Figure 4:**
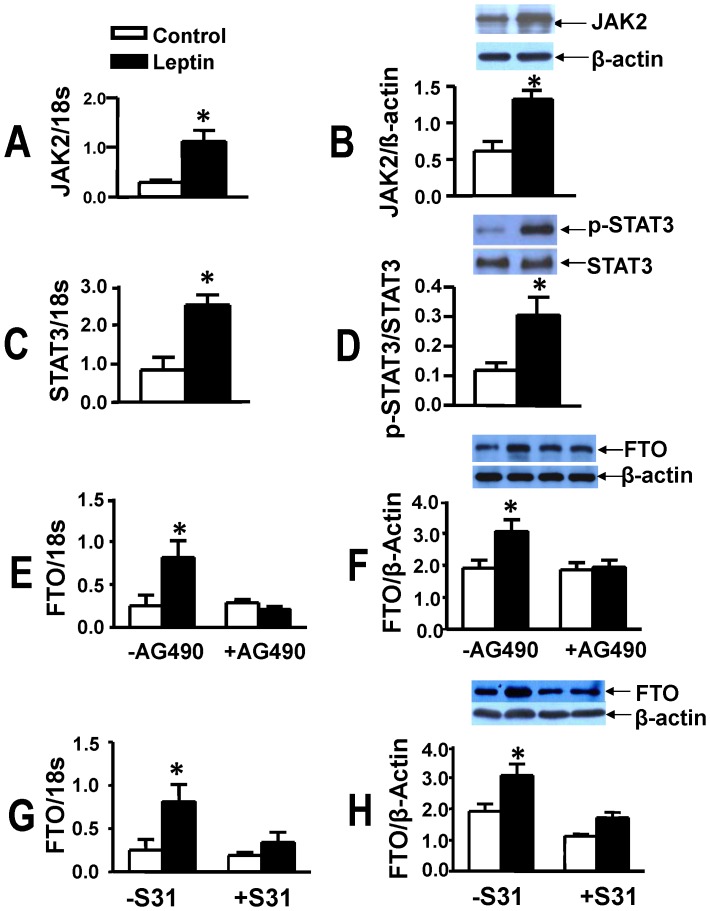
FTO upregulation is dependent on JAK2/STAT3 activity. Panels A to D show that leptin upregulates gene and protein expression of JAK2 (A,B), gene expression of STAT3 (C) and protein levels of p-STAT3 (D). Panels E to H show that leptin-induced upregulation of FTO is prevented by the JAK inhibitor AG490 (E,F) and the STAT3 inhibitor S31–201 (S31, G,H). Data are presented as mean+SEM, N = 8 *P<0.05 from control group not treated with leptin.

We next determined the potential cellular mechanisms by which leptin increases FTO abundance. As leptin exerts many of its effects *via* JAK2/STAT3 activation we determined the contribution of this system to FTO upregulation. Leptin produced a marked increase in JAK2 gene (Figure 4A) and protein expression (Figure 4B), STAT3 gene expression (Figure 4C) as well as protein levels of phosphorylated STAT3 (Figure 4D). Moreover, inhibition of either JAK2 with AG490 (Figure 4E and 4F) or STAT3 with S31–201 (Figure 4G and 4H) completely supressed leptin- induced upregulation in either gene or protein FTO expression.

**Figure 5:**
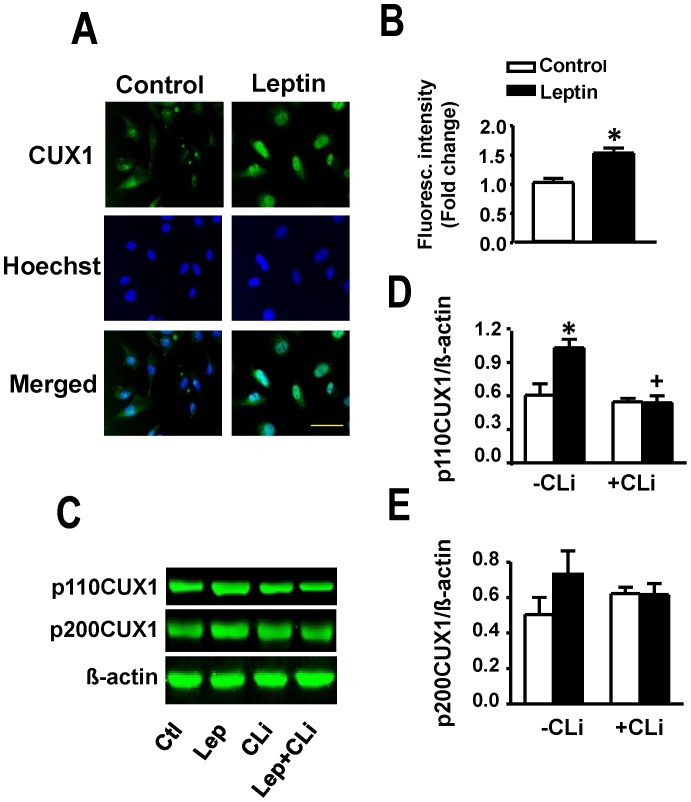
Upregulation of CUX1 by leptin. Panels A and B show that leptin increases CUX1 immunofluorescence intensity in cultured cardiomyocytes. Panels C to E demonstrate representative Western blots (C) and corresponding quantitative data (D, E) documenting increased protein expression of p110 CUX 1 with a non-significant effect on the p200CUX1 isoform. The increase in p110 CUX protein levels was abolished by the cathepsin L inhibitory peptide (CLi). Quantitative data are presented as mean +SEM. N = 7. *P<0.05 from control; ^+^P<0.05 from respective group in the absence of CLi (-CLi). Horizontal bar in bottom right images indicates 200 µm.

**Figure 6:**
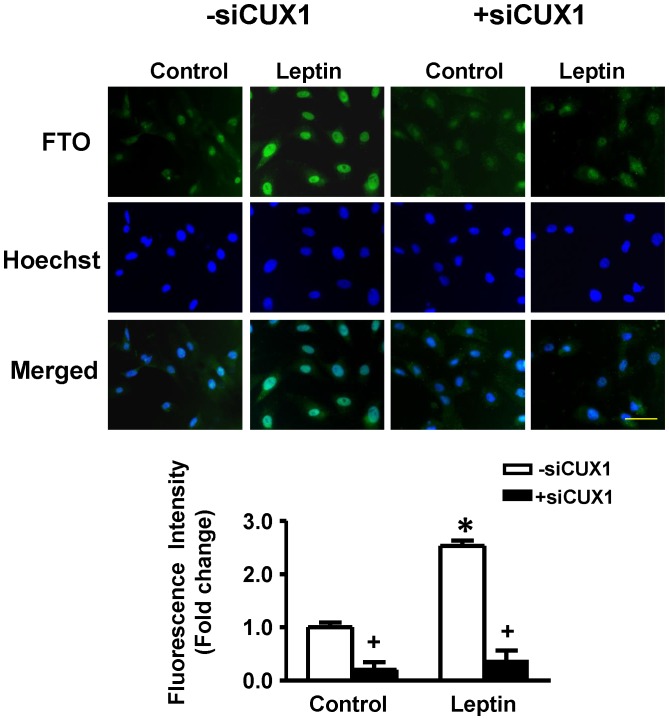
Suppression of CUX1 prevents leptin-induced FTO upregulation. Immunofluorescent images and corresponding quantified immunofluorescence intensity data showing that CUX1 siRNA (+siCUX1) supressed basal FTO expression and completely prevented the effect of leptin on FTO. Quantitative data are presented as mean +SEM. N = 7. *P<0.05 from respective control group; ^+^P<0.05 from respective group in the absence of siCUX1 (−siCUX1). Horizontal bar in bottom right images indicates 200 µm.

As CUX1 has been suggested as a possible regulator of FTO (please see Discussion), we sought to determine the effect of leptin on CUX1 and the potential role of the latter on leptin-induced FTO upregulation in cardiomyocytes. Total CUX1 as determined by immunofluorescence staining was significantly increased by leptin (Figure 5A and 5B). Individual CUX1 isoforms were measured using Western blotting (Figures 5C to 5E) which showed a significant increase in p110CUX1 protein expression with leptin treatment (Figure 5C and 5D). Although p200 CUX1protein levels tended to increase in response to leptin there were no significant differences between treatment groups (Figure 5C and 5E). The leptin-induced increase in p110 CUX1 protein expression was completely supressed by the cathepsin L peptide inhibitor (Figure 5C and 5D). siRNA directed at CUX1 produced a 80% reduction in basal FTO levels and a complete prevention of the ability of leptin to increase FTO expression (Figure 6).

**Figure 7:**
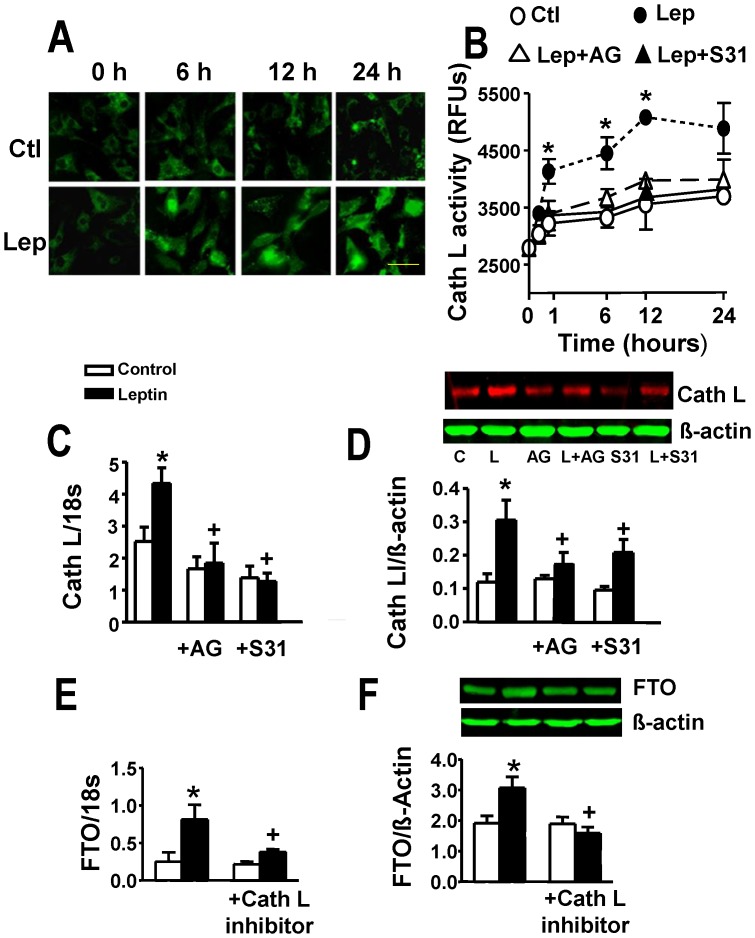
Regulation of cathepsin L (Cath L) expression and activity by leptin and the role of cathepsin L in leptin-induced FTO upregulation. Panel A shows time-dependent increases in cathepsin L immunofluorescence intensity following leptin (Lep) addition. Control (Ctl) group depicts myocytes not treated with leptin. Panel B provides evidence for leptin-induced increase in cathepsin L activity following leptin administration and its attenuation by the JAK2 inhibitor AG490 (AG) and STAT3 inhibitor S31–201 (S31). RFUs, relative fluorescence units. Panels C and D demonstrate increased gene and protein expression, respectively, of cathespsin L in cardiomyocytes treated with leptin and their prevention by inhibitors of JAK2 and STAT3. For representative Western blots L = leptin. Panels E and F demonstrate prevention of leptin-induced FTO gene and protein upregulation by the cathepsin L peptide inhibitor. Quantitative data are presented as mean ± SEM. N = 7. *P<0.05 from all other groups in panel B and respective control group in panels C to F. ^+^P<0.05 from the leptin alone group in panels C to F. For panels A and B, time refers to hours after leptin addition. Horizontal bar in bottom right images indicates 200 µm.

The possibility that leptin-induced increase in FTO levels reflected p200CUX1 proteolysis to p110CUX1 was studied by first determining whether leptin affects cathepsin L and then whether a cathespin L inhibitor exerts any influence on leptin-induced FTO elevation. Leptin produced marked and significant increases in cathepsin L gene and protein expression as well as activity which were abrogated by Jak2 and STAT3 inhibition (Figure 7A–7D). Moreover, a peptide cathepsin L inhibitor completely supressed the ability of leptin to increase both FTO gene and protein expression (Figure 7E and 7F).

**Figure 8:**
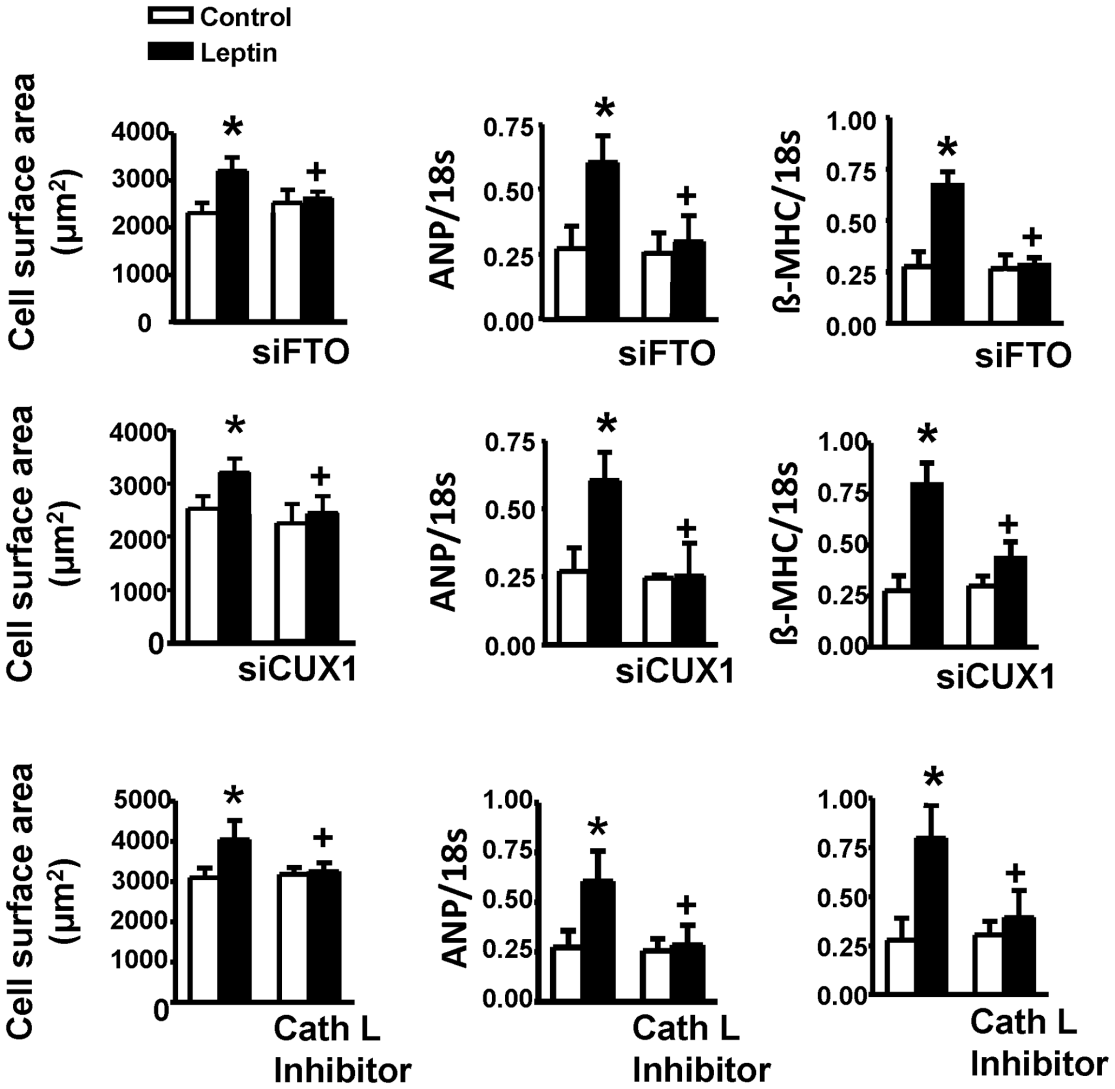
Hypertrophic responses to leptin with FTO, CUX1 and cathepsin L (Cath L) inhibition. Cell surface area is shown in the left row whereas expression of ANP and β-MHC are depicted in the middle and right rows, respectively. Note that the hypertrophic responses to leptin were abolished by all treatments. Data are presented as mean+SEM. N = 7. *P<0.05 from respective control values. ^+^P<0.05 from the leptin alone group.

Finally, we determined whether approaches aimed at supressing cathepsin L, CUX1 or FTO share an equal ability to attenuate the hypertrophic effect of leptin. As summarized in Figure 8, pharmacological inhibition of cathepsin L or knockdown of either CUX1 or FTO with siRNA prevented the hypertrophic response of cardiomyocytes to leptin. Moreover, these effects of leptin as well as its ability to activate JAK2/STAT3 were completely prevented by the leptin receptor antagonist SHLA (Table 2).

**Table 2:** Summary of effects of the leptin receptor antagonist SHLA on leptin induced JAK2/STAT3 activation increased gene and protein expression of p110CUX1 and cathepsin L and the hypertrophic response as determined by cell surface area and gene expression of ANP and β-MHC.

Parameter	Control	SHLA	Leptin	Leptin+SHLA
JAK2/18S	0.32±0.04	0.39±0.04	1.07±0.16*	0.34±0.03
JAK2/β-actin	0.63±0.07	0.59±0.06	1.38±0.13*	0.67±0.07
STAT3/18S	0.83±0.06	0.85±0.07	2.38±0.31*	0.94±0.10
p-STAT3/STAT3	0.12±0.02	0.14±0.03	0.29±0.04*	0.11±0.02
p110CUX1/β-actin	0.61±0.11	0.67±0.08	1.08±0.16*	0.62±0.07
Cathepsin L/18S	2.43±0.27	2.36±0.19	4.39±0.41*	2.48±0.33
Cathepsin L/β-actin	0.12±0.04	0.14±0.03	0.31±0.04*	0.12±0.05
CSA (µm^2^)	2178±183	2219±214	3094±314*	2339±346
ANP/18S	0.27±0.03	0.26±0.04	0.68±0.04*	0.30±0.05
β-MHC/18S	0.26±0.05	0.25±0.03	0.77±0.08*	0.28±0.04

The table summarizes the effect of the leptin receptor antagonist SHLA on various parameters including gene expression of JAK2, STAT3, cathepsin L, ANP and β-MHC (all indicated as ratios to 18S gene expression), protein levels of JAK2, p110CUX, cathepsin L (as ratios to β-actin) and P-STAT3 (as ratio to total STAT3) as well as cell surface area (CSA). SHLA completely abolished all responses to leptin. *P<0.05 from all other groups. N = 4.

## Discussion

In the present study we investigated the possible contribution of intracellular FTO to the pro-hypertrophic effect of leptin in cultured ventricular myocytes. Our study is in agreement with a previous report showing FTO expression in the heart [8] but extend those findings to clearly demonstrate that this occurs within the cardiomyocyte and, more specifically, that the expression is restricted within the nuclear compartment. Our study further demonstrates the ability of leptin to significantly increase nuclear FTO expression in cardiomyocytes as well as the involvement of FTO in the hypertrophic response, the latter observation based principally on the ability of FTO downregulation by siRNA to supress the hypertrophic response to leptin. Further, we show the critical link between the transcriptional factor CUX1 and regulation of FTO as well as an important role for CUX1 in leptin-induced hypertrophy. Lastly, our study implicates cathepsin L activation as a key contributor to the hypertrophic effect of leptin, likely through its ability to catalyze the proteolytic conversion of the transcriptional repressor p200CUX1 to the more stimulatory p110CUX1 isoform.

Although the precise role of leptin is still not completely understood especially as this pertains to cardiac pathology, its ability to directly induce a cardiomyocyte hypertrophic response has been shown by a number of investigators and the peptide appears to do so *via* a multiplicity of cell signalling pathways [11]–[13], [15]. FTO is a relatively recently identified gene discovered through obesity genome-wide associated studies (GWAS) [4]–[6] although, interestingly, the FTO gene was actually first identified in the so-called fused toes mutation in the mouse where it was found to be completely deleted [16]. The precise function of FTO is not well-established. As recently enunciated by Tung and Yeo [2], one of the reasons for this is that FTO was identified based on the “hypothesis-free” nature of GWAS research rendering the assignment of a functional role of FTO difficult. FTO however is likely of major importance in the regulation of growth development. Thus, FTO deletion in mice is associated with multiple phenotypes including postnatal growth retardation and decreased body mass due primarily to increased metabolic rate as food intake is unaltered in these animals [3]. However, overexpression of FTO produces increased food consumption resulting in obesity [7]. Virtually nothing is known concerning the role of FTO in cardiovascular disease. Although variations in the FTO gene have been shown to be associated with increased cardiovascular risk, this is thought to be reflective of the prevalence of obesity in these individuals [17], [18]. FTO functions as a nucleic acid demethylase which likely contributes to its regulation of body weight and adiposity although the nature of this involvement is not known [8].

As already alluded to in the introduction, FTO is expressed in the heart, and indeed in many other tissues thus suggesting tissue-specific multiple functions [8]. The cardiac function of FTO is completely unknown but we were intrigued by reports that leptin may regulate FTO in some tissues such as the hypothalamus where leptin has been shown to decrease FTO levels [10]. In the present report we show that leptin is a potent upregulator of FTO expression in cultured myocytes. Moreover, in rats treated with an antibody directed against the leptin receptor, a significant inhibition of FTO protein expression in the heart was observed suggesting that endogenous leptin regulates levels of cardiac FTO. The dose of the leptin receptor antibody (4 µg/kg bw) as well as the treatment regimen were identical to those which we have previously found to effectively supress the ability of acute leptin administration to enhance cardiac STAT3 phosphorylation [14]. This part of our study further showed that administration of a high fat diet and the resultant obesity failed to alter myocardial FTO expression. This finding was in contrast to our expectations since Tung et al. reported that 10 week treatment with a high fat diet (45% fat) in rats produced a 2.5 fold increase in FTO expression in the arcuate nuclear region of the hypothalamus [19]. These different results are unlikely due to variations in the experimental protocol as we used a relatively similar approach consisting of 12 week treatment with a 48% fat containing diet. However, the results may suggest that the regulation of FTO expression in the hypothalamus and heart reflect different mechanisms with the former demonstrating sensitivity to manipulation by dietary factors such as a high fat diet or perhaps by the resultant obesity and increased adiposity. In addition, it is possible that the modest elevation in plasma leptin concentrations seen in animals fed a high fat diet was insufficient to further upregulate cardiac FTO from basal levels thus suggesting that a greater degree of hyperleptinemia may be required to achieve this goal. In this regard, plasma leptin concentrations under conditions of severe obesity can exceed 100 ng/ml [20] and it would therefore be of importance to determine cardiac FTO expression under more pronounced hyperleptinemic conditions.

**Figure 9:**
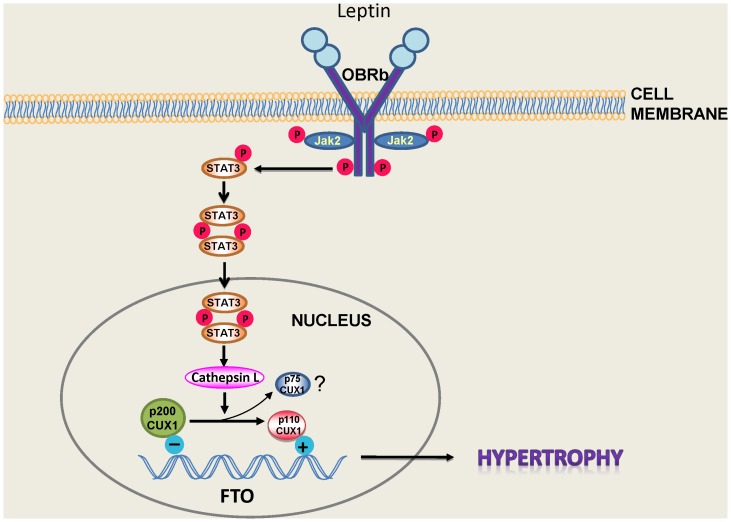
Proposed mechanism for leptin-induced JAK2/STAT3-dependent FTO upregulation. Based on this concept activation of the long form of the leptin receptor (OBRb) results in the dimerization and translocation of STAT3 into the nucleus where it activates the protease cathepsin L resulting in the proteolytic processing of p200CUX to the shorter p75 and p110 CUX1 proteins. The latter upregulates FTO resulting in the hypertrophic response. Please see Discussion for details.

Evidence from the literature suggests that leptin exerts its effects on the heart through multiple cellular mechanisms [11]–[13], [15]. In the present study we show for the first time that FTO plays a potentially important role in mediating the hypertrophic response to leptin and that leptin is an important regulator of FTO expression in the heart. Indeed the results presented here are the first to assign any functional role for cardiac FTO. As illustrated in Figure 9, we postulate a number of critical events underlying leptin-induced FTO upregulation which is dependent on post-receptor (OBRb) JAK2 upregulation resulting in phosphorylation of STAT3 and its dimerization and nuclear translocation where it regulates transcription of STAT3-dependent genes. This well-established leptin signalling pathway [21], [22] is not only important for the hypertrophic response to leptin but we have recently reported STAT3-dependent pro-apoptotic effects of leptin likely related to enhanced opening of the mitochondrial permeability transition pore [23].

CUX1 represents a family of transcriptional factors which exist as multiple isoforms. Among these, p200CUX1 functions as a transcriptional repressor whereas it can be proteolytically processed to the smaller p110CUX1 as well as p75CUX1 proteins, with p110 having the ability to function as both a transcriptional activator and repressor [24], [25]. Although p75CUX1 has been linked to various pathologies [25] we have been unable to identify expression of this short isoform in cardiomyocytes suggesting the possibility that p200CUX1 is preferentially or exclusively proteolytically converted to the p110CUX1 protein. As illustrated in Figure 6, we believe that this proteolytic conversion of p200CUX1 to p110CUX1 is an important contributor to leptin-induced FTO upregulation which is dependent on STAT3 activation as well as cathepsin L activity, based on several lines of evidence. First, the leptin-increased cathepsin L expression and activity were prevented by JAK2/STAT3 inhibition. Moreover, a cathepsin L inhibitory peptide abrogated the leptin-induced increase in p110CUX1 and FTO protein expression as well as the hypertrophic effect of the peptide, responses which were shared by administering CUX1 siRNA. We further propose that these mechanisms are importantly linked to the hypertrophic response to leptin since our results show that downregulation of either FTO or CUX1 or the pharmacological inhibition of cathepsin L can each abrogate the hypertrophic phenotype.

In summary our study shows a functional role for FTO in the ventricular myocyte as well as its regulation by leptin. To understand possible mechanisms underlying FTO regulation we concentrated our efforts on JAK2/STAT3-CUX1, based on existing literature regarding control of FTO in different tissues. However, and as alluded to above, the hypertrophic effects of leptin are complex and most likely involve multiple cell signalling processes reflecting the pleiotropic nature of the peptide. For example, we have proposed a critical role for the Ras homolog gene family, member A (RhoA)/Rho-associated, coiled-coil containing protein kinase (ROCK) pathway and the resulting changes in actin dynamics in mediating the hypertrophic effects of leptin and have recently reported that this pathway may also stimulate calcineurin activation and the resultant translocation into nuclei of the transcriptional factor NFAT, apparently *via* Ca^2+^ -independent mechanisms [26]. As such, it will be important to determine whether other leptin-related signalling pathways can regulate FTO in the heart. This study conducted on myocytes is the first to our knowledge to suggest a functional role for FTO in cardiovascular tissues. These findings warrant further studies into the role of FTO in cardiovascular regulation. The present results suggest that FTO involvement in cardiomyocyte hypertrophy may be specifically related to leptin since neither endothelin-1 nor angiotensin II exerted any effect on FTO protein abundance nor did FTO suppression mitigate the hypertrophic response to either agent. However, the hypertrophic program is extremely complex and multifaceted [27] and can be initiated by many other factors which were not studied in the present report. Thus, the possibility cannot be excluded that FTO plays a more widespread role in cardiovascular physiology and pathology thereby potentially representing an effective target for therapeutic intervention. Global FTO deletion results in severe metabolic abnormalities, growth retardation and earlier postnatal mortality, the latter occurring despite the fact that FTO is not required for embryonic development [3]. However, selective cardiac FTO inactivation could represent a very useful approach to further delineate a role for FTO in cardiac physiology or pathobiology.
